# Prediction of Compartment Syndrome after *Protobothrops mucrosquamatus* Snakebite by Diastolic Retrograde Arterial Flow: A Case Report

**DOI:** 10.3390/medicina58080996

**Published:** 2022-07-26

**Authors:** Yueh-Tseng Hou, Meing-Chung Chang, Ching Yang, Yu-Long Chen, Po-Chen Lin, Giou-Teng Yiang, Meng-Yu Wu

**Affiliations:** 1 Department of Emergency Medicine, Taipei Tzu Chi Hospital, Buddhist Tzu Chi Medical Foundation, New Taipei 231, Taiwan; brianann75@gmail.com (Y.-T.H.); b101101107@tmu.edu.tw (C.Y.); yulong0129@gmail.com (Y.-L.C.); taipeitzuchier@gmail.com (P.-C.L.); gtyiang@gmail.com (G.-T.Y.); 2 Department of Emergency Medicine, School of Medicine, Tzu Chi University, Hualien 970, Taiwan; 3 Division of Plastic Surgery, Taipei Tzu Chi Hospital, Buddhist Tzu Chi Medical Foundation, New Taipei 231, Taiwan; 05039@tzuchi.com.tw; 4 Division of Plastic Surgery, School of Medicine, Tzu Chi University, Hualien 970, Taiwan

**Keywords:** Post-snakebite compartment syndrome, diastolic retrograde arterial flow, *Protobothrops mucrosquamatus*, fasciotomy

## Abstract

Post-snakebite compartment syndrome (PSCS) is an uncommon but dangerous condition. Compartment syndrome-like symptoms after snakebite by *Protobothrops mucrosquamatus* (*P. mucrosquamatus*) are not effective in guiding fasciotomy. Objective evaluation of intracompartmental pressure measurements in patients with suspected PSCS is recommended. However, there is a lack of consensus regarding PSCS and indications for surgical intervention, including the threshold value of chamber pressure. In addition, intracompartmental pressure measurements may not be readily available in all emergency service settings. Measuring intracompartmental pressure in all snakebite patients for early diagnosis of PSCS is impractical. Therefore, identifying risk factors, continuous real-time monitoring tools, and predictive factors for PSCS are important. Sonography has proved useful in identifying the location and extension of edema after a snakebite. In this study, we attempted to use point-of-care ultrasound to manage PSCS in real-time. Here, we describe a rare case of snakebite from *P. mucrosquamatus*. PSCS was considered as diastolic retrograde arterial flow (DRAF) was noted in the affected limb with a cobblestone-like appearance in the subcutaneous area, indicating that the target artery was compressed. The DRAF sign requires physicians to aggressively administer antivenom to salvage the limb. The patient was administered 31 vials of *P. mucrosquamatus* antivenom, and fasciotomy was not performed. DRAF is an early sign of the prediction of PSCS.

## 1. Introduction

Snakebite venom may cause both systemic and locoregional effects. The use of antivenom can effectively control the systemic effects of venom and prevent fatal complications. However, locoregional effects after snakebites may occur more frequently than systemic events. Tissue necrosis, edema, blistering, and bruising are commonly present in the development of compartment syndrome, which is rare but may cause permanent physical deformities owing to residual sequelae after a snakebite. Post-snakebite compartment syndrome (PSCS) is an emerging condition characterized by an increase in intracompartmental pressure leading to subsequent neurovascular compromise and tissue necrosis. Typical signs and symptoms of PSCS are “6P”, including pain, paresthesia, pallor, paralysis, poikilothermia, and pulselessness. When PSCS is diagnosed, an early fasciotomy is necessary to prevent permanent muscle and nerve damage. However, PSCS is often hard to predict and diagnose after envenomation that may occur several hours or even days after a snakebite. Therefore, an effective and easy tool for detection and timely intervention may improve clinical outcomes. Herein, we present a case of *P. mucrosquamatus* bite, and try the use of a point-of-care ultrasound to facilitate clinical decisions for PSCS.

## 2. Case Presentation

**Figure 1:**
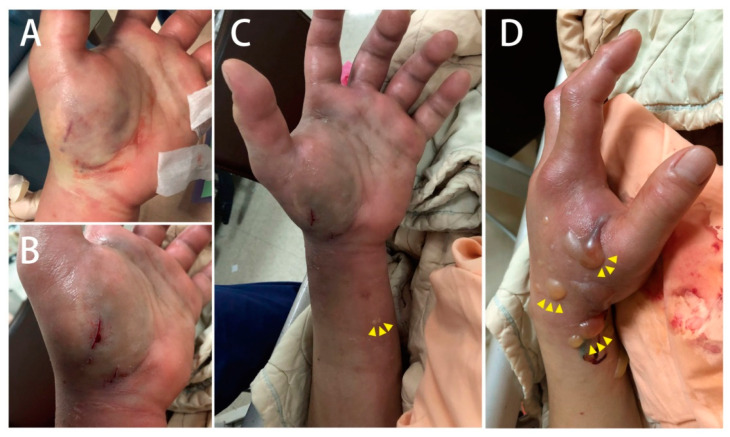
(**A**,**B**) Two puncture and laceration wounds on the left palm, (**C**) a tiny vesicle formed at the left wrist, and (**D**) progressed to multiple bullae after one hour.

**Figure 2:**
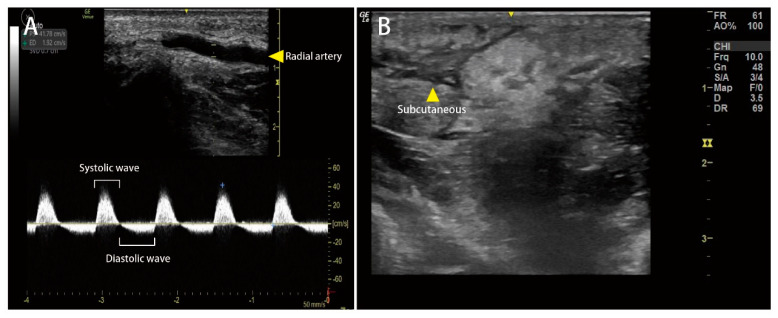
Point-of-care ultrasound (POCUS) findings in a patient bitten by *P. mucrosquamatus,* obtained one hour after the snakebite. (**A**) Diastolic retrograde arterial flow (DRAF) was noted in the affected limb, indicating that the target artery was compressed. (**B**) Cobblestone-like appearance (yellow arrowhead) located in the subcutaneous area.

**Figure 3:**
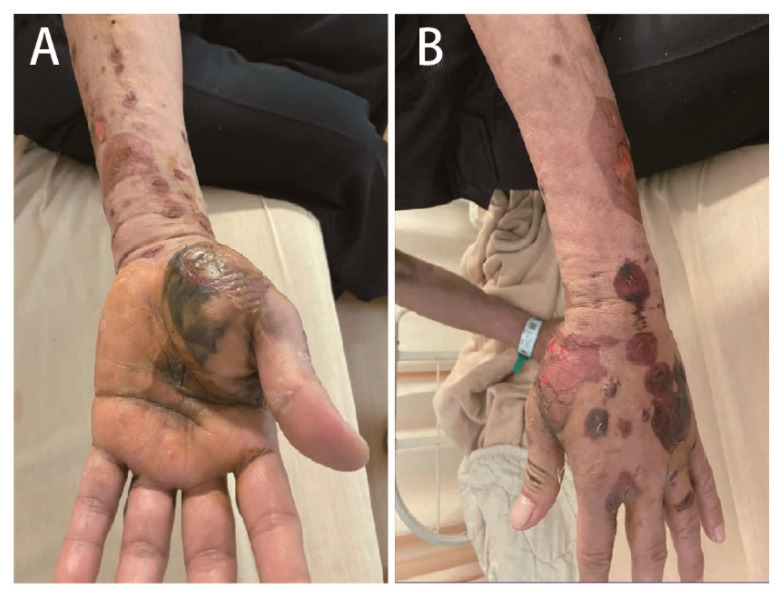
On day 7 of the snakebite, the post-snakebite compartment syndrome in the affected limb improved by the administration of antivenom and aspiration of multiple bullae to eliminate venom depots.

A 61-year-old male with an unremarkable medical history was admitted to the emergency department with a snakebite injury on his left wrist one hour after the snakebite. The patient confirmed that the snake was *Protobothrops mucrosquamatus (P. mucrosquamatus)*. Physical examination revealed a temperature of 36.2 °C, blood pressure of 225/100 mmHg, heart rate of 94 beats/min, and Glasgow Coma Scale score of E4V5M6. His skin had two puncture wounds and two laceration wounds on the left palm with severe swelling, local erythema, and ecchymosis. There was no numbness, weakness, slurred speech, or dyspnea. Detailed laboratory analyses are shown in Table 1. Only D-dimer levels were elevated. No significant coagulopathy or thrombocytopenia was noted. Four vials of *P. mucrosquamatus* antivenom were administered in a timely manner in the emergency department to prevent compartment syndrome. After one hour, a tiny vesicle formed at the left wrist and progressed to multiple bullae (Figure 1). Although the capillary refilling time of the distal fingers was less than two seconds, sonographic vascular peripheral arterial assessment of the left distal radial artery showed diastolic retrograde arterial flow (DRAF), reflecting the compression of the target artery (Figure 2). The results of peripheral vascular Doppler ultrasound were incompatible with capillary refilling time. However, a high risk for compartment syndrome was suspected. Therefore, *P. mucrosquamatus* antivenom was aggressively administered every two hours and the patient was admitted for close observation. Multiple bullae were aspirated to eliminate the venom depots. In the ward, distal radial arterial Doppler pulse and capillary refilling time were regularly checked to detect compartment syndrome. The left-hand swelling and ecchymosis condition improved with antivenom administration (Figure 3). A follow-up laboratory analysis revealed significantly elevated D-dimer levels. Finally, he was administered 31 vials of *P. mucrosquamatus* antivenom.

## 3. Discussion

PSCS is an uncommon but dangerous condition. The incidence rate of surgical intervention after Crotalinae snakebites is high [1]. Crotaline snakebites, including *Protobothrops mucrosquamatus* and *Trimeresurus stejnegeri*, are common venomous snakebites that account for the majority of envenoming events in Taiwan. Envenoming by Crotalinae snakebites leads to severe tissue edema and pain in the affected extremities, mimicking compartment syndrome [2,3]. In a study [3], 54.6% (53/97 patients) patients were admitted for observation during the acute period. Of these patients, 75.4% (40/53 patients) were followed up for 48–72 h and 18.8% (10/53 patients) were followed up for 96–120 h due to compartment syndrome-like symptoms. Only three patients finally received fasciotomy. All of these patients responded to 20% mannitol and antivenom therapy that relieved the symptoms and decreased compartment pressure. A descriptive study of snakebite patients in northern Taiwan from 2009 to 2016 showed 50% (63/125) patients were bitten by *P. mucrosquamatus*, and the surgical rate was high, up to 23.8% [1].

There are several reasons to explain why compartment syndrome-like symptoms commonly occur in *P. mucrosquamatus* snakebites. First, extensive swelling and persistent ecchymosis are common after snakebite by *P. mucrosquamatus,* even with antivenom therapy, and mimic compartment syndrome. Second, snake venom with phospholipase A2 and metalloproteinase may directly destroy soft tissue by breaking down muscle fibers and type IV collagen in capillaries. Compartment syndrome-like symptoms after snakebite by *P. mucrosquamatus* are directly caused by snake venom without increasing compartment pressure [4,5,6]. Finally, the host response to snake venom may contribute to local tissue damage, including the formation of neutrophil extracellular traps [7]. Therefore, compartment syndrome-like symptoms after snakebites caused by *P. mucrosquamatus* are not effective in guiding fasciotomy.

The ischemic symptoms and signs of PSCS usually occur in the late period and should not be relied on for the early diagnosis of PSCS. Therefore, to identify risk factors, continuous real-time monitoring tools and predictive factors are important. A classification of snakebite wounds was proposed to determine PSCS in the absence of compartment pressure measurement devices [3]. In groups 0 and 1, there was only a bite trace and local pain less than 2 cm in the extremity diameter. In group 2, there were mild systemic symptoms and 2–4 cm of the extremity diameter. Patients in group 3 had more than 4 cm of extremity diameter and were exhibited edematous cold and pulselessness. Patients with higher group snakebite wounds, especially in group 3, should be monitored for a minimum of 24 h and should be monitored for PSCS if necessary using the intracompartmental pressure monitoring system.

Laboratory analysis was also an effective tool to predict PSCS [8,9]. In a study [9], 6.6% (9/136) patients developed PSCS and the authors found a significant increase in the white blood cell (WBC) count, segment form, aspartate aminotransferase level, and alanine aminotransferase level in the PSCS group. In multivariate analysis, the level of WBC count (Cut-off value: 11650/μL with sensitivity of 66.7% and specificity of 83.6%) and AST (Cut-off value: 33.5 U/L with sensitivity of 85.7% and specificity of 78.9%) were risk factors for PSCS. These markers reflect inflammatory or cytokine reactions during the host response [10]. Acute hemolysis and severe necrosis of the skeletal muscles in the PSCS population increased AST levels. In symptomatic snakebite patients, elevated WBC or AST levels should be considered in the development of PSCS. Our case did not show leukocytosis or AST on day one. The elevated AST levels were observed after four days.

Objective evaluation of patients with suspected PSCS, such as intracompartmental pressure measurement is recommended. However, there is a lack of consensus regarding PSCS and indications for surgical intervention, including the threshold value of chamber pressure [11,12]. Some studies advocate close follow-up and fasciotomy if there is clinical suspicion. In a population prone to trauma-induced compartment syndrome, early fasciotomy is the most efficacious. Lately, fasciotomy has resulted in similar rates of limb salvage but a higher risk of infection [11]. Fırat et al. [13] suggested that early fasciotomy in the PSCS population also resulted in better recovery than late fasciotomy. In early fasciotomy, local edema and toxic symptoms are rapidly diminished by enhancing the circulation of the extremities and clearing the toxins. Controversially, some studies have suggested postponing fasciotomy is necessary as fasciotomy may cause complications. Few patients require fasciotomy after snakebite due to PSCS, and earlier fasciotomy increases morbidity [14,15,16]. Most compartment syndrome-like symptoms regress with adequate antivenom therapy. If intracompartmental pressure progressively increases to over 55 mm Hg, fasciotomy should be considered [3]. In addition, intracompartmental pressure measurements may not be readily available in all emergency service settings. Measuring intracompartmental pressure in all snakebite patients for early diagnosis of PSCS is impractical. In our case, the plastic surgeon used compartment syndrome-like symptoms to assess PSCS, and surgeons were concerned that fasciotomy may impair the function of the affected extremity. Therefore, we attempted to use an objective method via point-of-care ultrasound to manage PSCS.

Several studies have highlighted the benefit of sonography in identifying the location and extension of edema after snakebites [2,17,18]. Wood et al. [17] measured the dimensions of the subcutaneous tissues and the deep muscle compartment at the affected limb compared with the unaffected limb to calculate an expansion coefficient for early detection of PSCS. However, it is difficult to establish the absolute criteria for the diagnosis of PSCS based on morphometric analysis [19]. Ho et al. [2] proposed a theory that increased intracompartmental pressure in the PSCS population compromised blood circulation, leading to tissue hypoxia and nerve injury. When the development of PSCS influences vascular compliance and resistance, diastolic retrograde arterial flow (DRAF) can be observed in the affected artery, reflecting a restriction of the compartment space [20,21]. This phenomenon has been demonstrated by Mc Loughlin et al. [21]. The authors found that greater percentages of DRAF were detected in healthy volunteers under a pressure cuff with 40 mm Hg, equal to the diastolic blood pressure and mean arterial pressure. The appearance of DRAF seems to be an ideal tool for the serial evaluation of PSCS development. However, Ho et al. [2] analyzed 17 snakebite patients bitten with *P. mucrosquamatus* and sonography showed subcutaneous edema in all patients, but DRAF was not detected in any of them. In our patient, diastolic retrograde arterial flow (DRAF) was noted in the affected limb with a cobblestone-like appearance in the subcutaneous area, indicating that the target artery was compressed. Early signs of PSCS were detected using POCUS, which made emergency physicians change their therapeutic strategies.

The venom of *P. mucrosquamatus* includes 61 distinct proteins belonging to 19 families, including snake venom metalloproteinase (SVMP; 29.4%), C-type lectin (CLEC; 21.1%), snake venom serine protease (SVSP; 17.6%), and phospholipase A2 (PLA2; 15.9%) [22]. The key treatment for PSCS from *P. mucrosquamatus* bites is the administration of horse-derived antivenom, produced by the Centers for Disease Control, R. O. C. (Taiwan). The antivenom of *P. mucrosquamatus* is a bivalent F(ab)2 fragment antivenom with a neutralization effect of over 1000 Tanaka units per vial [23,24,25]. Based on the current consensus on *P. mucrosquamatus* bite management, approximately 1–4 vials of antivenom are recommended for patients with *P. mucrosquamatus* snakebites [25,26,27,28]. In our case, four vials of antivenom were administered due to the progression of compartment-like symptoms 8 h after the snakebite. However, POCUS showed DRAF, suggesting early PSCS. The antivenom of *P. mucrosquamatus* was administered every two hours and the interval was increased to four hours if PSCS improved. The patient was administered 31 vials of *P. mucrosquamatus* antivenom for limb salvage. Although fasciotomy was not performed, we believed that DRAF was an early sign to reflect compression of the target artery. DRAF was detected even with local edema in the subcutaneous area. Severe subcutaneous edema may also cause DRAF if the snakebite wound is in the distal limb. However, DRAF is an early sign reflecting targeted arterial compression but is not specific for PSCS. Partial compression of the targeted artery may also cause DRAF, as demonstrated by McLoughlin et al. [21]. DRAF is an early and effective sign to alert emergency physicians to consider PSCS in patients with snakebites of *P. mucrosquamatus*.

## 4. Conclusions

Herein, we present a case of *P. mucrosquamatus* bite. Snakebite and PSCS were considered due to DRAF and the cobblestone-like appearance at the affected limb, reflecting the compression of the target artery. The appearance of DRAF requires physicians to aggressively administer antivenom for limb salvage. The patient was administered a total of 31 vials of *P. mucrosquamatus* antivenom, and a fasiotomy was not performed. We believe that DRAF is an early sign of PSCS.

## Figures and Tables

**Table 1:** The laboratory evaluation of the patient on day 1 and 4.

Variables	Normal Range	Patient Data		Variables	Normal Range	Patient Data	
		Day1	Day4			Day1	Day4
White cell count	3.9–10.6 × 109/L	7.38	8.80	Creatinine	0.04–0.09 mmole/L	0.82	0.83
Band form Neu.	0–3%	0.0	0.0	Sodium	136–145 mmole/L	137	138
Segment form Neu.	45–70%	64.2	71.6	Potassium	3.5–5.1 mmole/L	4.3	3.6
Lymphocytes	25–40%	27.1	20.3	CRP	<1.0 mg/dL	0.3	1.34
Eosinophils	1–3%	3.9	1.9	Glucose	3.9–5.6 mmole/L	138	---
Monocytes	2–8%	4.5	6.0	ALT	16–63 U/L	10	46
Hemoglobin	13.5–17.5 g/dL	14.7	13.8	AST	15–37 U/L	8	138
Platelet counts	150–400 × 109/L	274	308	BUN	7–25 mg/dL	14	13
PT	8.0–12.0 s	10.3	---				
APTT	23.9–35.5 s	28.2	---				
FDP-Ddimer	0–500 µg/L	782.44	3102.06				
INR	----	0.99	---				

Neu.: neutrophils; PT: Prothrombin time; APTT: Partial thromboplastin time; CRP: C-reactive protein; AST: Aspartate aminotransferase; ALT: Alanine aminotransferase.

## Data Availability

Not applicable.
